# Pathophysiology of Heart Failure: A Role for Peripheral Blood Mononuclear Cells Mitochondrial Dysfunction?

**DOI:** 10.3390/jcm11030741

**Published:** 2022-01-29

**Authors:** François Sauer, Marianne Riou, Anne-Laure Charles, Alain Meyer, Emmanuel Andres, Bernard Geny, Samy Talha

**Affiliations:** 1 University of Strasbourg, Translational Medicine Federation of Strasbourg (FMTS), Faculty of Medicine, Team 3072 “Mitochondria, Oxidative Stress and Muscle Protection”, 11 rue Humann, 67000 Strasbourg, France; francois.sauer@chru-strasbourg.fr (F.S.); marianne.riou@chru-strasbourg.fr (M.R.); Anne.Laure.charles@unistra.fr (A.-L.C.); alain.meyer1@chru-strasbourg.fr (A.M.); emmanuel.andres@chru-strasbourg.fr (E.A.); samy.talha@chru-strasbourg.fr (S.T.); 2 University Hospital of Strasbourg, Physiology and Functional Exploration Service, 1 Place de l’Hôpital, 67091 Strasbourg, France; 3 Internal Medicine, Diabete and Metabolic Diseases Service, University Hospital of Strasbourg, 1 Place de l’Hôpital, 67091 Strasbourg, France

**Keywords:** heart failure, peripheral blood mononuclear cell, PBMC, mitochondria, oxidative stress, pathophysiology

## Abstract

Heart failure (HF) is a leading cause of hospitalization in patients aged more than 65 years and is associated with high mortality rates. A better comprehension of its physiopathology is still needed, and, in addition to neurohormonal systems and sodium glucose co-transporter 2 modulations, recent studies focus on the mitochondrial respiration of peripheral blood circulating cells (PBMCs). Thus, cardiovascular metabolic risk factors and cellular switch with an increased neutrophil/lymphocytes ratio might favor the decreased PBMC mitochondrial respiration observed in relation with HF severity. PBMCs are implicated in the immune system function and mitochondrial dysfunction of PBMC, potentially induced by their passage through a damaged heart and by circulating mitoDAMPs, which can lead to a vicious circle, thus sustaining negative cardiac remodeling during HF. This new approach of HF complex pathophysiology appears to be a promising field of research, and further studies on acute and chronic HF with reduced or preserved LVEF are warranted to better understand whether circulating PBMC mitochondrial function and mitoDAMPs follow-ups in HF patients might show diagnosis, prognosis or therapeutic usefulness.

## 1. Introduction

Heart failure (HF) is a clinical syndrome defined by reduced cardiac output and/or elevated intracardiac pressures at rest or during exercise. It is characterized by typical symptoms, such as breathlessness, ankle swelling and fatigue, which may be accompanied by signs (elevated jugular venous pressure, pulmonary crackles and peripheral edema) caused by structural and/or functional cardiac abnormalities (ESC 2021). In Europe, there are 15 million HF patients and 120,000 new cases are reported each year in France. The mortality is about 10% and HF is the leading cause of hospitalizations in patients aged more than 65 years (177,000/year). Sympathetic nervous, renin–angiotensin–aldosterone and cardiac natriuretic system implications are well-known and their inhibition is the cornerstone of chronic HF management, which has been recently implemented with sodium glucose co-transporter 2 modulators, even in HF-preserved ejection fractions (HFpEFs) up to 60% [1,2,3,4,5]. Treatment of chronic HF is relatively consensual when the left ventricular ejection fraction (LVEF) is reduced (<40%), and management of acute HF is based on diuretics, triggering factor specific treatment and the early initiation of the recommended chronic therapy. Nevertheless, new targets are needed and might be identified through an improvement in HF pathophysiology knowledge. Interestingly, recent studies in HF have focused on inflammation and systemic oxidative stress potentially related to the mitochondrial dysfunction of peripheral circulating cells.

In this view, the mitochondrial respiratory function of peripheral blood mononuclear cells (PBMCs) (in extenso lymphocytes and monocytes) is easily available. These cells are involved in many inflammatory diseases, including those driven by ischemia-reperfusion episodes, which play a key role in cardiovascular alterations [6]. Whether their mitochondrial dysfunction could be used as a diagnostic, severity and/or prognosis biomarker in cardiovascular diseases is under evaluation, but the major role of mitochondria in cell energy and reactive oxygen species (ROSs) production support such a hypothesis. Thus, adequate ROS levels in terms of the amount and duration of secretion are considered as useful signaling factors. On the other hand, ROS secreted in excess and not buffered by antioxidants can lead to protein, lipid, and DNA damage and tissue dysfunctions. Accordingly, ROSs are increased in ischemic muscles and in pathological myocardium during HF [7,8], and circulating blood ROSs can also be interesting biomarkers of disease severity.

This brief review aims to report and discuss the data investigating the evolution and potential usefulness of PBMC mitochondrial respiration in the setting of human HF. This is a current challenge and a better comprehension of HF pathophysiology might lead to significant advances in HF management. Indeed, impaired PBMC mitochondrial respiration might be implicated in the development of inappropriate immune system responses and negative cardiovascular remodeling during HF.

## 2. Potential Relationships between PBMC, Mitochondrial Function and HF

T-cell recruitment and myocardial infiltration is well-described during ischemic and non-ischemic HF [9], with specific and enhanced abnormal interactions with cardiac antigens as compared to controls [10]. During infection, heart transplant or ischemic injury, these cells infiltrate the heart, in association with a disrupted self-tolerance of cardiac antigen. Altogether, these cellular crosstalks negatively affect cardiac remodeling and function [11]. Monocytes also play a central role, being recruited during a myocardial injury by a cell–cell interaction [12,13]. They favor fibroblasts proliferation, and in fine cardiac fibrosis. Moreover, neutrophils are described as a potential “weapon” for sterile inflammation and can lead to abnormal cardiac remodeling and HF development [14].

However, cellular mechanisms and bioenergetic dysfunctions leading to these pathological implications are poorly known, and PBMC mitochondrial function could play a significant role in this immune-related disorder during HF. Indeed, these easily available biomarkers are essential for adaptive immune system responses to organ injuries, and the recent data support an impaired mitochondrial respiration of PBMC in the setting of HF.

The first data were reported by Li et al. [15] who described a global decrease in mitochondrial respiratory function in 25 early stage, asymptomatic HFpEF patients, as compared to 24 controls (Table 1). The early stage asymptomatic patients were defined by cardiac remodeling (septal hypertrophy and/or a delayed relaxation) and the presence of a cardiometabolic risk factor (essential hypertension, dyslipidemia and type 2 diabetes). Moreover, the high sensitivity C-reactive protein, interleukin-6 (IL-6) and tumor necrosis factor-α (TNF-α), were significantly higher, and superoxide dismutase—a major antioxidant actor—transforming superoxide anion into hydrogen peroxide, was reduced in HF patients.

Then, Shirakawa et al. [16] observed in 31 compensated chronic HF patients with reduced EF (HFrEF) < 35% that the activities of several mitochondrial respiratory chain complexes were altered. Thus, complexes I + II and the maximal electron transfer system with complexes I + II and II alone were significantly reduced in functional class NYHA III (16 patients) versus the NYHA I or II groups (15 patients), (*p* < 0.05). This suggests that impaired PBMC mitochondrial respiration is related to the degree of severity of the disease. At the same time, mitochondrial ROSs were significantly higher in the NYHA III group than in the NYHA I or II groups.

Recently, data from 19 stage-D HFrEF patients also showed an impairment of maximal respiration as compared to the controls, with the basal oxygen consumption rate tending to be lower [17].

The causes potentially involved in PBMC-reduced mitochondrial respiration during HF are yet to be determined, but several hypotheses might be raised. Both cardiovascular metabolic risk factors and cellular switch might play a role.

Cardiovascular metabolic risk factors strongly associated with HF development [18] and chronic inflammation [19] might participate in such a depressed PBMC mitochondrial respiration during HF. Thus, a recent study showed that LDL-c is negatively associated with global mitochondrial respiration [20]. Altered mitochondrial respiration is also described in hypertension-related renal, cardiac and vascular disease, and age-related cardiac changes [21,22]. These abnormalities in PBMC could participate in systemic endothelial dysfunction [23].

**Figure 1:**
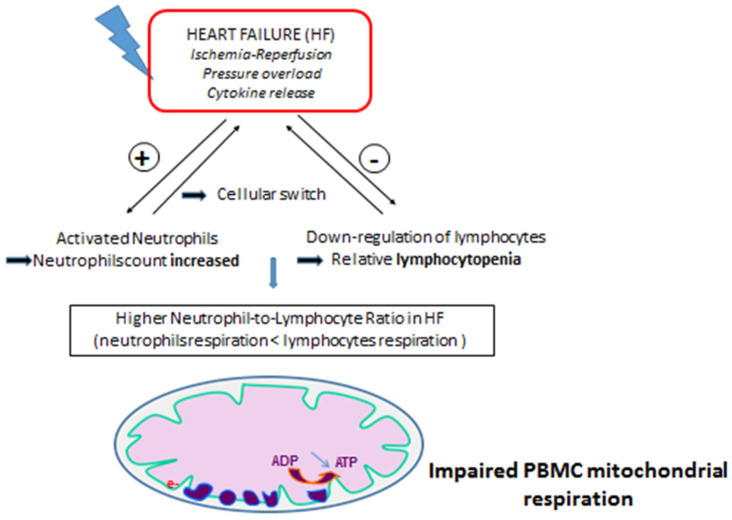
A cellular switch with increased neutrophils and reduced lymphocyte counts can lead to a global decrease in PBMC mitochondrial respiration during heart failure. PBMC: peripheral blood mononuclear cell. ADP: adenosine diphosphate. ATP: adenosine triphosphate.

Interestingly, there is a cellular switch (Figure 1) in the white cell count during HF development that could participate in the global decrease in mitochondrial respiration. Indeed, neutrophils are activated and increased and could facilitate cardiomyocytes’ apoptosis [24], whereas there is a progressive and relative lymphocytopenia due to the inflammation and down-regulation of the immune system [25]. This cellular switch in HF patients could lead to a decrease in global PBMC mitochondrial respiration, since neutrophils seem to have a minimal contribution to the oxygen consumption rate and cellular bioenergetics, as compared to lymphocytes and monocytes [26]. Indeed, studies show that the neutrophils-to-lymphocytes ratio (NLR) is increased in acute and chronic HF patients and is associated with frequent congestive HF decompensation, hospitalization readmission and one-year overall mortality [24,27,28,29].

## 3. Potential Mechanisms Involved in Impaired Mitochondrial Respiration of PBMC in HF

Mitochondrial respiration is essential for PBMC homeostasis and its dysfunction could lead to numerous inflammatory responses in various diseases. On a mouse model with a defective mitochondrial function in CD4+ T lymphocytes, impaired mitochondrial respiration lead to lysosome dysfunction. Thus, this alteration exhibited in vitro and in vivo inflammatory responses with a higher level of cytokines (Interferon-γ) and T-cell proliferation [30]. During the first 7 days of a septic shock, a recent study found a global increase in the global mitochondrial respiration capacity and a paradoxical decrease in ATP-synthase activity [31]. These changes might be a mitochondrial adaptation to acute systemic stress. On the other hand, in a chronic state, such as chronic HF, the decreased non-adaptive mitochondrial respiration could have a negative impact. Indeed, Zhou et al. showed that the impaired mitochondrial respiration of the PBMC of patients with HF resulted in increased secretion of proinflammatory cytokines [17]. Furthermore, altered mitochondrial respiration is also associated with less effective T-cell migration [32].

Accordingly, a link between immune cell activation and immune cell metabolism has been demonstrated. In general, the activation of immune cells requires a rise in energy and most articles suggested that activated monocytes and T and B lymphocytes undergo metabolic reprogramming and an increased glucose uptake and glycolysis. On the contrary, long-living immune cells, such as macrophages or regulatory T cells, enhance oxidative phosphorylation and beta-oxidation to produce their energy [33]. The role of the different respiratory complexes is variable between the different types of immune cells, but there is a lot of evidence to suggest that modified mitochondrial respiration is involved in inflammatory responses.

Concerning HF, both the “proximity theory” and activation of the immune system cells by circulating factors arising from the heart might account for the relationships between PBMC and HF.

**Figure 2:**
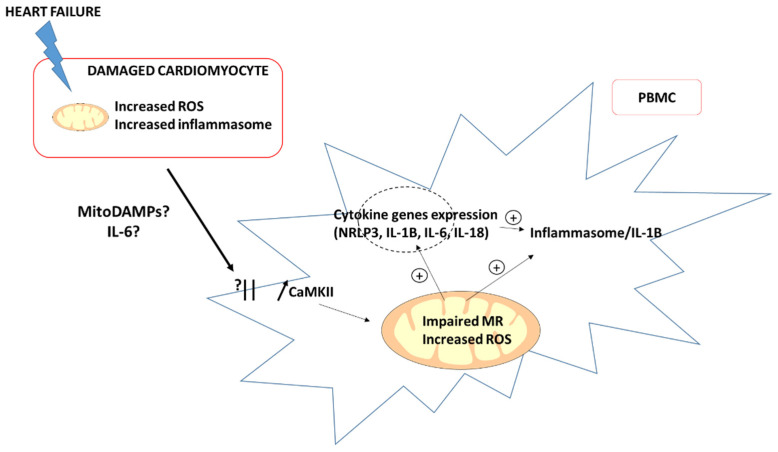
The “proximity theory”: communication between the damaged cardiomyocytes and PBMC in the heart. CaMKII: calmoduline kinase II; MitoDAMPs: mitochondrial damaged-associated molecular patterns; MR: mitochondrial respiration and ROS: reactive oxygen species.

The “proximity theory” proposes that PBMCs are activated during their passage through the heart (Figure 2). Indeed, during non-ischemic stress (pressure overload, angiotensin II exposure), there is a communication between the immune system near the myocardium and damaged cardiomyocytes, especially through the calmoduline kinase II (CaMKII)/NRLP3 pathway (NRLP3 for nucleotide-binding, leucine-rich repeat and pyrin-domain-containing 3) [34]. The initial mitochondrial dysfunction in the cardiomyocytes leads to increased cellular stress, which further activates the inflammasome/interleukin-1β (IL-1β) pathway and immune cells’ recruitment [35]. Moreover, circulating IL-6 is increased and could participate in chronic HF development [36,37]. Interestingly, Zhou et al. [17] suggested that IL-6 acts as a signal, connecting the mitochondrial function and inflammation in PBMCs, leading to mitochondrial respiration impairment by inhibiting complex I activity.

The second hypothesis might be the circulation of factors arising from the heart and activating the immune system cells. Mitochondrial DNA (mtDNA) and oxidative stress are likely to be involved as circulating factors. Confirming such a hypothesis, Zhou et al. [17] demonstrated that mtDNA extracted from myocardial damaged mitochondria elicited an impairment of the maximal oxygen consumption rate in healthy PBMC. Moreover, in this study, mitochondrial ROS and inflammatory cytokine gene expression (NRLP3, IL-1B, IL-6 and IL-18) were increased.

**Figure 3:**
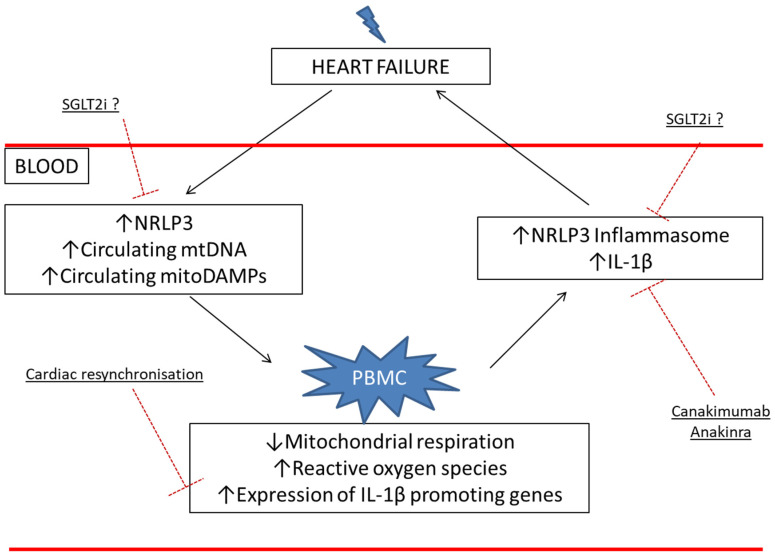
Potential vicious circle and therapeutic options: the alteration of PBMC mitochondria could exacerbate and/or sustain cardiac dysfunction and disease severity. NRLP3: nucleotide-binding, leucine-rich repeat and pyrin-domain-containing 3; PBMC: peripheral blood mononuclear cells and SGLT2i: sodium glucose co-transporter 2 (SGLT2) inhibitors.

Since ROS and mitochondrial dysfunction activate the immune system and inflammation, especially through NRLP3 activation [38], circulating mtDNA and extensively mitochondrial damage-associated molecular patterns (mitoDAMPs) could be systemic vectors of mitochondrial dysfunction in the heart, and can induce an impairment of PBMC mitochondrial respiration, which in turn might activate the immune system and inflammation (Figure 3). The impairment of mitochondrial respiration then leads to an ROS increase, which promotes the NLRP3 inflammasome activation [39]. One consequence is a higher production of IL-1β, a cytokine known for its negative effects on myocardium [40,41].

Interestingly in the CANTOS trial, the IL-1β blockade by canakinumab showed an HF-related decreases in hospitalization and mortality rates [42]. Similar to the IL-1β blockade by anakinra, Van Tassell et al. found an improvement of LV pressures as assessed by echocardiography after 12 weeks of treatment [43]. Corroborating a potential circulating factor and IL-1β implication, Bilchik et al. demonstrated that cardiac resynchronization therapy (CRT) in HF responder patients reduced the expression of inflammation-promoting genes related to IL-1 β in PBMC [44].

Recently, 2021 ESC guidelines on acute and chronic HF recommended the use of sodium glucose co-transporter 2 inhibitors (SGLT2i) as a safe and efficient first-choice treatment in HF with a reduced left ejection fraction (<40%) (1). One potential systemic effect is the reduction in inflammation through the inhibition of the NLRP3 inflammasome, independently of glucose lowering [45]. Accordingly, in the myocardium of diabetic rats, SGLT inhibition seems to increase mitochondrial respiration [46] and thus energy production. Moreover, these treatments improved the ketone level, a substrate of cardiac mitochondrial respiratory chains, in order to produce a high level of adenosine triphosphate (ATP) [47]. Other proposed mechanisms are the inhibition of HIF-1α negative effects, especially on the biogenesis of mitochondria [48,49] and on the activation of inflammatory macrophages [50]. Thus, these medications could have an interesting impact on PBMC mitochondrial respiration in HF, through the metabolic and non-metabolic pathways. Measurements of the mitochondrial respiration of PBMC from HF patients exposed to SGLTi will be useful to further investigate such issues.

Taken together, one can propose that PBMC mitochondrial impairment can lead to a vicious circle, finally altering the myocardium by activating fibrosis signaling and cardiac remodeling [51]. Accordingly, therapeutics studies targeting various cytokines (NLRP3, IL-1 β and IL-6) [52,53,54] showed promising results and support the need for a better comprehension of the activation of the immune system during HF.

## 4. Conclusions and Current Challenges

This new approach of HF complex pathophysiology appears to be a promising field of research. PBMCs are an easily available cell population implicated in the immune system function, and their mitochondrial dysfunction potentially induced by circulating mitoDAMPs may lead to a vicious circle sustaining cardiac dysfunction.

Nevertheless, most HF patients, whether with a reduced or preserved EF, have multiple comorbidities, and future studies trying to control comorbidities might be an important way to tease out the specific effects of these comorbidities, as they may produce differential molecular pathways to achieve the same vicious cycle of inflammation. Accordingly, individuals with a normal cardiac function but with type II diabetes, asthma exacerbation or pulmonary hypertension presented with an increased PBMC mitochondrial function [55,56,57].

Multisite clinical trials, in which large numbers of HF patients are studied with standardized PBMC mitochondrial function analysis measured alongside different blood biomarkers and clinical outcomes, and using linear regression modeling to control for individual comorbid conditions, are therefore warranted to better understand whether the circulating PBMC mitochondrial functions and mitoDAMPs follow-up in HF patients might show diagnosis, prognosis or therapeutic usefulness.

## Figures and Tables

**Table 1:** Studies investigating PBMC mitochondrial respiration during heart failure.

	Study 1 [15]. (Li et al., 2015)	Study 2 [16]. (Shirakawa et al., 2019)	Study 3 [17]. (Zhou B et al., 2020)
Type	Comparative (vs. controls)	Comparative according to NYHA	Comparative (vs. controls)
Population	25 chronic HFpEF (>50%) asymptomatic patients	31 chronic HFrEF (<35%) patients	19 stage-D HFrEF (<35%) patients
Mitochondrial respiration	Impaired	Impaired according to NYHA	Impaired
Mitochondrial ROSs in PBMC	Decreased antioxidant capacity	Increased	Increased

NYHA: New York Heart association. HF: heart failure. rEF: reduced ejection fraction. PBMC: peripheral blood mononuclear cell. ROS: reactive oxygen species. pEF: preserved ejection fraction.
